# Expressions of ezrin, ERK, STAT3, and AKT in tongue cancer and association with tumor characteristics and patient survival

**DOI:** 10.1002/cre2.293

**Published:** 2020-04-13

**Authors:** Masaharu Noi, Ken‐ichi Mukaisho, Shoko Murakami, Shinya Koshinuma, Yoshisato Machida, Masashi Yamori, Takahisa Nakayama, Takao Ogawa, Yusuke Nakata, Takeshi Shimizu, Gaku Yamamoto, Hiroyuki Sugihara

**Affiliations:** ^1^ Department of Oral and Maxillofacial Surgery Shiga University of Medical Science Ōtsu Japan; ^2^ Division of Molecular Diagnostic Pathology, Department of Pathology Shiga University of Medical Science Ōtsu Japan; ^3^ Department of Otorhinolaryngology Shiga University of Medical Science Ōtsu Japan

**Keywords:** AKT, ERK, the 5‐year survival rate, tongue squamous cell carcinoma

## Abstract

**Background:**

Ezrin, ERK, STAT3, and AKT are proteins that are overexpressed in various types of cancer, although their expressions in tongue cancer has received less focus. This study aimed to address associations between the expression levels of these proteins and with characteristics of the tumor and patient survival.

**Methods:**

We performed immunohistochemical staining of ezrin, ERK, STAT3, and AKT in tumors from patients with tongue carcinoma in situ (CIS, n = 17) and tongue squamous cell carcinoma (SCC, n = 46). Statistical differences between the SCC versus the CIS cohorts were estimated by calculations of bivariate odds ratios of low versus high expression of the proteins. Fisher's exact tests were used to appraise interassociations between the proteins, as well as expression levels versus patient and tumor characteristics. Survival based on Kaplan–Meier statistics in combination log‐rank tests were used to address potential effects of the patient and tumor characteristics versus 5‐year survival rate.

**Results:**

The relative high: low expression of all four proteins in the two cohorts differed, and particularly ERK was markedly overexpressed in the SCC versus the CIS cohort (odds ratio = 45.3, *p* < .01). The relative high: low expression each protein versus patient and tumor characteristics; showed associations between AKT expression and T stage (*p* = .002) plus node metastases (*p* = .12), and between ERK expression and drinking (*p* = .01) and smoking history (*p* = .01). There was no significant difference observed between ERK and the three other molecules, nor any significant difference between the degree of expression of each protein and the 5‐year disease‐specific survival rate.

**Conclusion:**

Ezrin, ERK, STAT3, and AKT appear to be involved in the progress from carcinoma in situ in the tongue into squamous cell carcinoma. ERK in particular is overexpressed, suggesting that ERK may be a novel therapeutic target for preventing tongue cancer.

## 1 INTRODUCTION

Oral tongue squamous cell carcinoma (OTSCC) accounts for approximately 1.5% of all cancer cases and is an aggressive cancer that is frequently associated with a poor prognosis (Ganly, Patel, & Shah, 2012). In Japan, the number of patients with oral cancer has been increasing annually. In oral cancer cases, the tongue is the most frequently observed site of cancer development (Japan Society for Head and Neck Cancer Registry Committee, 2003). It has been estimated that 6–7% cases of tongue cancer occur in patients aged <40 years (Garavello, Spreafico, & Gaini, 2007).

The tongue has a rich lymphatic network, which makes it the site that is most frequently associated with cervical metastasis of cancer at other sites in the oral cavity. The presence of nodal metastasis in the neck is the most important prognostic factor. In addition, clinicopathological prognostic factors, including TNM stage, grade, and the depth of tumor invasion, are essential in predicting the course of disease and in the determination of appropriate treatment methods. Despite considerable advances in diagnostic and therapeutic techniques, patients with OTSCC have a poor prognosis, with an estimated 5‐year overall survival rate of only 56% (Bettendorf, Piffko, & Bankfalvi, 2004). We previously reported that ezrin is overexpressed in OTSCC and plays a vital role in tongue cancer cell growth, migration, and invasiveness via the E‐cadherin/b‐catenin complex and cadherin switch (Saito et al., 2013).

Theocharis et al. have reported that ERK is overexpressed in tongue cancer cells and infiltrating lymphoid cells and is correlated with important clinicopathological parameters of patient management and prognosis. Therefore, ERK expression and activation in tumor cells and infiltrating lymphocytes is considered to be a useful prognostic marker in tongue cancer (Theocharis et al., 2014). Moreover, Stat3 is involved in the motility, metastasis, and progression in human lingual SCC (Zhao et al., 2013). The activation of AKT is an important prognostic factor for disease‐free survival of patients with OTSCC regardless of the stage and lymph node status and can be used as a predictive marker for second primary tumors and recurrences (Massarelli et al., 2005).

Many studies have reported that ezrin, ERK, STAT3, and AKT are associated with the development of tongue cancer cells and with the prognosis of patients with OTSCC. However, for OTSCC, there are few reports assessing the association between these molecules and patient survival rate; moreover, the associations between ezrin, ERK, STAT3, and AKT expressions and clinicopathological features have been analyzed using the same sample.

The purpose of this study was to analyze the association between the expression of each molecule and the survival rate of patients with OTSCC, and the clinicopathological features of OTSCC using immunohistological staining.

## 2 MATERIALS AND METHODS

### 2.1 Patients

This study included 63 patients who received treatment for OTSCC from the Dental Oral Surgery or Otolaryngology departments at the Shiga University of Medical Science Hospital between January 2005 and December 2014. Data regarding patient sex and age, tumor size/extent, presence of regional lymph node metastases, distant metastases, and clinical stage were collected from medical records. Tumor staging was performed according to the TNM classification of the International Union Against Cancer. Histological differentiation was defined according to the World Health Organization classification. Before surgery, all patients underwent a physical examination, computed tomography (CT), magnetic resonance imaging, and ultrasonography and positron emission tomography/CT examination to examine the primary tumor and cervical lymph nodes. Following primary partial glossectomy with unilateral or bilateral cervical lymph node dissection, the final histopathology report confirmed that all patients had negative surgical margins. Formalin‐fixed paraffin‐embedded block specimens of carcinoma in situ (CIS) (17 cases) and invasive SCC of the tongue (46 cases), surgically removed at the Shiga University of Medical Science Hospital, were examined in this study. This study was approved by the Ethics Committee of the hospital (approval no. 27–78).

### 2.2 Immunohistochemical staining

Sequential sections (3‐μm thick) were prepared from the paraffin blocks of maximum cut surface specimens for each case. These samples were immunohistochemically stained for ezrin, ERK, AKT, and STAT3. The intensity of staining was classified on a 4‐point scale: 0, 1+, 2+, and 3+, with 0 and 1+ indicating low levels and 2+ and 3+ indicating high levels. The degree of staining was compared between CIS and SCC. The antibodies used were anti‐ezrin rabbit antibody (1:100, #3145; Cell Signaling, Danvers, MA), anti‐p44/42 MAP kinase mouse antibody (1:500, #4696; Cell Signaling), anti‐AKT mouse antibody (1:250, #2920; Cell Signaling), and anti‐STAT3 mouse antibody (1:600, #9139; Cell Signaling.

Immunostaining was performed using a Discovery XT Automated IHC Stainer with a Ventana DABMap Detection kit (no. 760–124; Ventana Medical System, Oro Valley, AZ). Each step of the Ventana DABMap detection kit procedure was optimized for the Discovery XT instrument; conditions including the dilutions of each antibody were established before measurements. In all cases, the antigen was activated by heating.

### 2.3 Statistical analysis

Descriptive statistics as used to describe characteristics of the subjects in the SSC and CIS cohorts and subjected to chi‐square statistics to assess differences with regard to categorical demographic variables. Bivariate odds ratios were estimated on basis of low versus high expression of respectively, ezrin, ERK, STAT 3, and AKT in the SCC versus the CIS cohort. The high/low expressions of ERK versus high/low expressions of ezrin, STAT 3 and AKT were appraised by use of Fisher's exact test. The associations between the high/low expressions of ezrin, ERK, STAT 3, and AKT versus patient and tumor characteristics were analyzed in the SCC cohort using Fisher's exact test. Finally, the 5‐year disease‐specific survival (DSS) rate was calculated by using the Kaplan–Meier method. Log‐rank tests were applied to assess differences in survival rates as a function of high/low expressions of the target molecules. *p* < .05 was considered to indicate a statistically significant difference.

## 3 RESULTS

### 3.1 Patient characteristics

The clinical characteristics of patients and the comparison of CIS and SCC are shown in Table 1. The mean age was 65.5 years (range, 33–86 years) for SCC cases and 67.6 years (range, 44–87 years) for CIS cases. Among the SCC cases, T1 was the highest at 24 cases (52.2%), and 11 cases (23.9%) showed lymph node metastasis. Histological differentiation was most common in 33 cases (71.8%) in Well. In terms of smoking history, the proportion of SCC cases with or without a smoking history was almost the same, whereas, more CUS cases had a smoking history. In terms of drinking history, SCC and CIS cases were more likely to be absent. There was no significant difference between CIS and SCC for sex, smoking history, or drinking history.

**Table 1:** Patient and tumor characteristics of patients with tongue carcinoma in situ (CIS) and tongue squamous cell carcinoma (SCC)

Characteristics	SCC	CIS	*p*‐value
	(n = 46) (%)	(n = 17) (%)	
Age (years)			
Range	33–86	44–87	
Mean	65.5	67.6	
Gender			
Male	23 (50.0)	11 (64.7)	.3962
Female	23 (50.0)	6 (35.3)	
T stage			
Tis	—	17 (100)	
T1	24 (52.2)	—	
T2	12 (26.1)	—	
T3	1 (2.1)	—	
T4	9 (19.6)	—	
Node metastases			
No	35 (76.1)	17 (100)	
Yes	11 (23.9)	—	
Clinical stage			
Stage I	23 (50.0)	—	
Stage II	10 (21.8)	—	
Stage III	2 (4.3)	—	
Stage IV	11 (23.9)	—	
Histological differentiation			
Well	33 (71.8)	—	
Moderate	12 (26.1)	—	
Poor	1 (2.1)	—	
Smoking history			
No	25 (54.3)	7 (41.2)	.4048
Yes	21 (45.7)	10 (58.8)	
Drinking history			
No	29 (63.0)	12 (70.6)	.7673
Yes	17 (37.0)	5 (29.4)	

*Note:* Chi‐square statistics.

### 3.2 Comparison of the expression of each molecule between CIS and SCC

Immunostaining was performed on CIS and SCC, and the degree of expression of the molecule was evaluated. The results are shown in Table 2.

**Table 2:** High versus low expression of the proteins Ezrin, ERK, STAT 3, and AKT in tongue cancer

Expression of molecules	Ezrin		ERK		STAT3		AKT	
	Low	High	Low	High	Low	High	Low	High
CIS (n = 17)	13	4	16	1	14	3	16	1
SCC (n = 46)	7	39	12	34	14	32	22	24
Odds ratio	18.1		45.3		10.7		17.5	
*p*‐value	*p* < .01		*p* < .01		*p* < .01		*p* < .01	

*Note:* Fischer exact test statistics.

**Figure 1:**
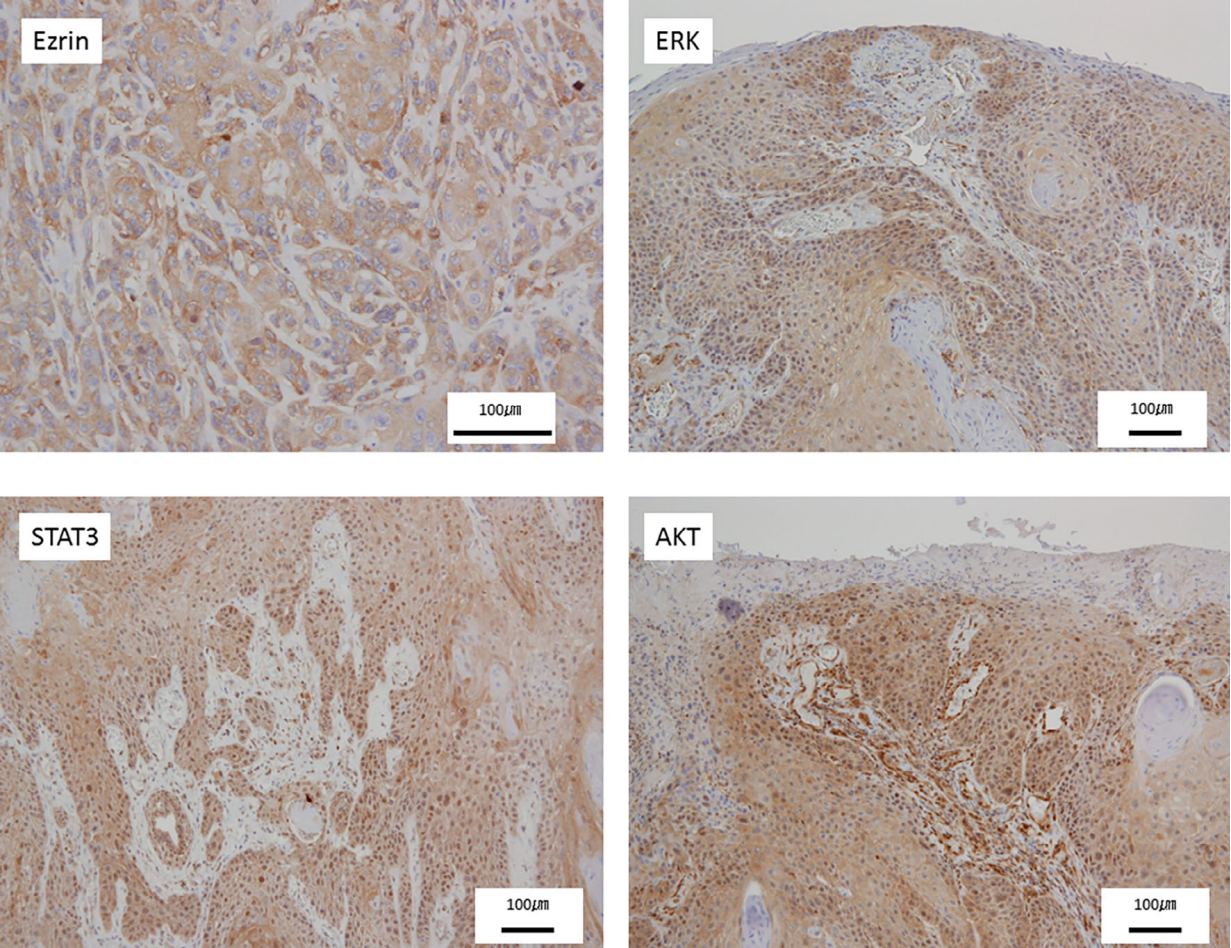
Ezrin‐, ERK‐, STAT3‐, and AKT‐stained images of human tongue cancer tissue (scale bar: 100 μm). Brown color represents cells positive for protein, as visualized by DAB staining

In cases of high expression, ezrin expression was particularly high in the cell membrane. Stat3 was expressed highly in the cytoplasm and nucleus of cancer cells. ERK and AKT were mainly expressed in the cytoplasm of cancer cells, whereas AKT was expressed at high levels in the invasive front of tumor foci (Figure 1). The degrees ezrin, ERK, STAT 3, and AKT expressions were significant different between CIS and SCC cases. It was suggested that the molecules evaluated in this study are involved in the development of CIS to SCC in the tongue. Among them, ERK exhibited a low correlation (OR = 45.3), which was present at low levels in 16 cases and high levels in three cases for CIS, and low levels in 12 cases and high levels in 34 cases for SCC.

ERK was followed by ezrin (OR = 18.1), AKT (OR = 17.5), and STAT 3 (OR = 10.7), with minimal difference in correlation strength between ezrin and AKT. Fisher's exact test revealed that ERK expression is likely to be associated with ezrin expression, but as with the other molecules, no significant difference was observed (Table 3).

**Table 3:** High versus low expression of the ERK versus the proteins Ezrin, STAT 3, and AKT in tongue cancer

		Ezrin		STAT3		AKT	
		Low	High	Low	High	Low	High
ERK	Low	4	8	5	7	8	4
	High	3	31	9	25	14	20
*p*‐value		.06		.47		.18	

*Note:* Fischer exact test statistics.

### 3.3 Associations between the expression of each molecule and tumor characteristics in SCC

The degree of expression of each protein was appraised relative to patient and tumor characteristics in the SCC cohort (Table 4). For age, sex, and histological differentiation, there was no significant difference for any molecule.

**Table 4:** High versus low expression of the proteins Ezrin, ERK, STAT 3, and AKT in tongue cancer versus patient and tumor characteristics of patients with tongue squamous cell carcinoma (SCC

SCC characteristics	Ezrin		ERK		STAT3		AKT	
	Low	High	Low	High	Low	High	Low	High
Age (years)								
>65	6	26	8	24	6	26	11	21
≤65	4	19	6	17	9	14	11	12
*p*‐value	1.000		1.000		.1282		.4055	
Gender								
Male	7	24	11	20	6	25	10	21
Female	3	21	3	21	9	15	12	12
*p*‐value	.4856		.0666		.2216		.2675	
T stage								
T1 + T2	6	34	9	31	13	27	21	19
T3 + T4	4	11	5	10	2	13	1	14
*p*‐value	.4339		.4926		.1923		.0019	
Node metastases								
No	5	34	8	31	12	27	19	20
Yes	5	11	6	10	3	13	2	14
*p*‐value	.1342		.3060		.5103		.0150	
Histological differentiation								
Well	9	33	8	34	12	30	16	26
Moderate/poor	1	12	6	7	3	10	6	7
*p*‐value	.4214		.0710		1.000		.7481	
Smoking history								
No	4	25	3	26	9	20	10	19
Yes	6	20	11	15	6	20	12	14
*p*‐value	.4901		.0118		.5578		.4196	
Drinking history								
No	3	29	4	28	8	24	13	19
Yes	7	16	10	13	7	16	9	14
*p*‐value	.0751		.0131		.7621		1.000	

*Note:* Fischer exact test statistics.

The degrees STAT3 and AKT expressions tended to increase as the T stage progressed. Fisher's exact test revealed that AKT expression was likely to be associated with the T stage, with a significant difference observed (*p* < 0.05). Fisher's exact test showed that ERK expression was likely to be associated with ezrin expression, but as with the other molecules, no significant difference was observed. The degrees of ezrin and AKT expressions tended to increase as node metastases progressed. Fisher's exact test revealed that AKT expression was likely to be associated with node metastases, with a significant difference observed (*p* < .05). Therefore, it was suggested that AKT is associated with the growth and metastasis of tongue cancer cells.

In terms of smoking history, the degree of ERK expression tended to decrease as smoking history was reported, indicating that ERK expression was likely to be associated with smoking history, and a significant difference was observed (*p* < .05). Similarly, for drinking history, ERK expression level tended to decrease as a drinking history was recognized (*p* < .05). This suggested that ERK is strongly related to smoking history and drinking history.

### 3.4 Associations between the expression of each molecule and the 5‐year DSS

Univariate analysis using the log‐rank test and the Kaplan–Meier method demonstrated that ezrin and STAT3 expression levels, and AKT were likely to be associated with 5‐year DSS, but no significant difference was observed between cases with high levels and low levels.

**Figure 2:**
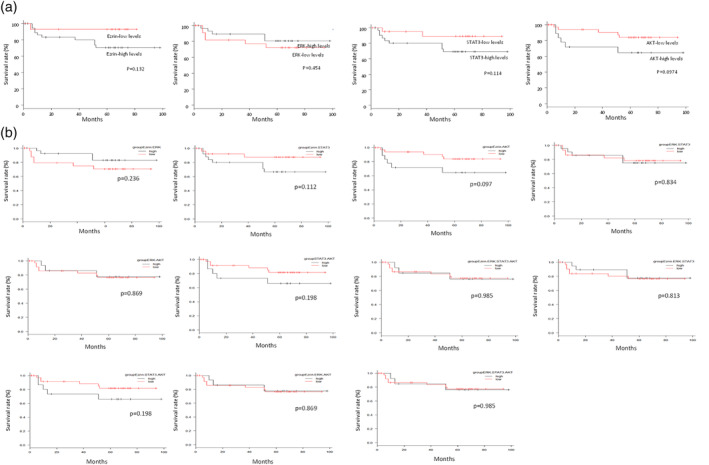
Kaplan–Meier 5‐year disease‐specific survival (DSS) rate evaluated according to the expression levels of Ezrin, ERK, STAT3, and AKT. (a) There was no significant difference between high levels and low levels of various proteins. For ERK, a high level had a higher 5‐year DSS rate than a low level. (b) There were no significant differences between the high and low levels of the various proteins

By contrast, patients with low levels of ERK had a lower 5‐year DSS than those with high levels of ERK (Figure 2a).

Although the 5‐year DDS was analyzed between the groups by combining ezrin, ERK, STAT3, and AKT, no significant difference was recognized between high levels and low levels (Figure 2b).

## 4 DISCUSSION

The results of the present study suggest that ezrin, ERK, STAT 3, and AKT are involved in the development from tongue CIS to tongue SCC, that AKT is associated with T stage, and that ERK is associated with alcohol and smoking habits. By contrast, the results indicated that there was no significant difference between the molecules and the 5‐year DSS, which was not associated with the survival rate.

Serine/threonine protein kinase AKT, an important target of the phosphoinositide‐3‐kinase/AKT signaling pathway, is abnormally activated in various types of tumor, including those in head and neck cancer (Amornphimoltham et al., 2004) (including OTSCC) (Massarelli et al., 2005).

AKT is able to phosphorylate and regulate a number of proteins associated with cell metabolism, apoptosis, proliferation, and differentiation, thereby inhibiting the apoptosis of tumor cells and promoting the proliferation of tumor cells.

The activation of AKT is associated with the progression of squamous epithelial cells from the normal epithelium to dysplasia, and then to invasive carcinoma (Amornphimoltham et al., 2004); in the results of the present study, AKT expression was also significantly high in cases with an advanced T stage. It was confirmed that AKT was associated with the progression of OTSCC.

OTSCC is associated with numerous factors, including age, geographical location, and family history. Tongue cancer is also associated with habits including smoking and alcohol abuse. Although each is an independent risk factor, these two factors may potentially synergize and increase the risk of developing cancer. In an epidemiological survey targeting Caucasian men, the relative risk of HNSCC was 34 times higher for people who had these habits than for those without tobacco and alcohol use, seven times higher for tobacco only, and three times higher for alcohol only (Osei‐Sarfo, Tang, Urvalek, Scognamiglio, & Gudas, 2013).

The nicotine contained in tobacco activates ERK, promotes cancer cell proliferation and angiogenesis through the nicotinic acetylcholine receptor, and is involved in tumor growth, and the tongue influenced by alcohol and tobacco is reported to have increased ERK expression in vivo (Osei‐Sarfo et al., 2013). However, our results showed that ERK expression tended to be lower for cases with a positive smoking history and drinking history, contrary to the results of previous reports. Although the factors remain unknown, the associations among smoking, alcohol, and ERK require further investigation regarding OTSCC.

ERK is a downstream component of the signal module activated by Raf serine/threonine kinase. In cancer, the ERK/MAPK signal is abnormally activated by the upstream activation by epidermal growth factor receptor (epidermal growth factor receptor) and Ras GTPases (small guanosine triphosphatase), and it is involved in promoting cancer cell proliferation, survival, and metastasis (Roberts & Der, 2007). The ERK expression is reported to cause resistance to radiation therapy and chemotherapy in malignant melanoma (Smalley, 2003).

The inhibition of ERK signaling inhibits tumor growth in various types of cancer, including gastric cancer (Wu et al., 2013), renal cell carcinoma (Fang et al., 2013), and rhabdomyosarcoma (Renshaw et al., 2013). We have also revealed that the activation of ERK in tongue cancer cell lines cultured three‐dimensionally using silica fiber is associated with the formation of invadopodia (Noi et al., 2018).

However, ERK has not been reported to be associated with survival in patients with prostate cancer (Han et al., 2017), and even in the present study, although the 5‐year DSS was high in cases with high levels of ERK compared with those with low levels, there was no significant difference between them.

Ezrin expression in breast cancer (Wang et al., 2009), endometrial cancer (Ohtani et al., 1999), uterine cancer (Kobel et al., 2006), uveal melanoma (Ilmonen, Vaheri, Asko‐Seljavaara, & Carpen, 2005), and soft tissue sarcoma (Weng, Ahlén, Aström, Lui, & Larsson, 2005) is associated with low survival rate. STAT3 has been reported to be associated with the survival rate of patients with nonsmall cell lung cancer (Sun et al., 2018) and AKT has been associated with survival rate in esophageal cancer (Shan, Chen, Wang, Wang, & Fan, 2017). However, in the present study, as with ERK, there was no significant difference between ezrin, STAT3, or AKT and 5‐year DSS. Therefore, it was suggested that these molecules are not associated with survival in OTSCC. It has been reported that ERK serves an important role in the preinvasive stage of oral epithelial tumors (Degen, Natarajan, Barron, Widlund, & Rheinwald, 2012); compared with the association of molecules including ezrin, STAT3, and AKT and the development from tongue CIS to OTSCC, ERK was found to have the highest correlation (OR = 45.3), consistent with previous reports.

There are been various reports on the association between ERK and ezrin. For example, Ezrin‐mediated early metastatic survival was partially dependent on the activation of MAPK, but not AKT in osteosarcoma (Khanna et al., 2004). In addition, the phosphorylation of ERK was reduced in ezrin‐KD cells, and activation of the ERK/MAPK pathway may partially attenuate the Ezrin‐mediated suppression of cell invasiveness in esophageal SCC (Xie et al., 2009). In addition, the inhibition of ezrin reduces the interaction with EGFR and reduces the level of phosphorylation of ERK and STAT3, which are downstream target genes of EGFR (Saygideğer‐Kont et al., 2016).

In terms of the relationship between AKT and ERK, there are reports that AKT can inhibit ERK signaling and cause a shift in cancer cellular responses from cell cycle arrest to proliferation (Zimmermann & Moelling, 1999), and that ERK and AKT pathways can collaborate to maintain cell viability (Dent, 2014). However, the exact intersects between ERK and ezrin, AKT, and STAT3 are not fully understood. In the present study, no significant difference was observed following comparison of the degree of the expression of ERK and other molecules in the same specimen.

Currently, with the evidence that ERK signaling is involved in cell proliferation, cell survival, and metastasis, numerous laboratories have focused on the development of ERK inhibitors for cancer treatment (Roberts & Der, 2007). In the future, ERK may be a target for prevention and control of the progress of OTSCC.

## CONFLICTS OF INTEREST

The authors declare no potential conflict of interest.

## AUTHOR CONTRIBUTIONS

Masaharu Noi carried out nearly all experiments and wrote the paper. Ken‐ichi Mukaisho designed the experiments and contributed advice during the writing of the paper. Shoko Murakami helped with experiments. Shinya Koshinuma, Yoshisato Machida, Masashi Yamori, Takahisa Nakayama, Takao Ogawa, Yusuke Nakata, Takeshi Shimizu, Gaku Yamamoto, and Hiroyuki Sugihara participated in discussions during the experiments and writing of the paper and offered valuable advice.
